# Impact of an Online Medical Internet Site on Knowledge and Practice of Health Care Providers: A Mixed Methods Study of the Spinal Cord Injury Rehabilitation Evidence Project

**DOI:** 10.2196/jmir.3453

**Published:** 2014-12-23

**Authors:** Janice J Eng, Vanessa K Noonan, Andrea F Townson, Caroline E Higgins, Jess Rogers, Dalton L Wolfe

**Affiliations:** ^1^Department of Physical TherapyUniversity of British ColumbiaVancouver, BCCanada; ^2^International Collaboration on Repair Discoveries (ICORD)Vancouver, BCCanada; ^3^Rehabilitation Research ProgramGF Strong Rehab CentreVancouver, BCCanada; ^4^Rick Hansen Institute (RHI)Vancouver, BCCanada; ^5^Division of Physical Medicine & RehabilitationUniversity of British ColumbiaVancouver, BCCanada; ^6^Centre for Effective PracticeToronto, ONCanada; ^7^Institute of Health PolicyManagement and EvaluationUniversity of TorontoToronto, ONCanada; ^8^Lawson Health Research InstituteLondon, ONCanada; ^9^Western UniversityLondon, ONCanada

**Keywords:** Internet, e-health, spinal cord injury, rehabilitation, evidence-based practice

## Abstract

**Background:**

It is not known whether ongoing access to a broad-based Internet knowledge resource can influence the practice of health care providers. We undertook a study to evaluate the impact of a Web-based knowledge resource on increasing access to evidence and facilitating best practice of health care providers.

**Objective:**

The objective of this study was to evaluate (1) the impact of the Spinal Cord Injury Rehabilitation Evidence (SCIRE) project on access to information for health care providers and researchers and (2) how SCIRE influenced health care providers' management of clients.

**Methods:**

A 4-part mixed methods evaluation was undertaken: (1) monitoring website traffic and utilization using Google Analytics, (2) online survey of users who accessed the SCIRE website, (3) online survey of targeted end-users, that is, rehabilitation health care providers known to work with spinal cord injury (SCI) clients, as well as researchers, and (4) focus groups with health care providers who had previously accessed SCIRE.

**Results:**

The online format allowed the content for a relatively specialized field to have far reach (eg, 26 countries and over 6500 users per month). The website survey and targeted end-user survey confirmed that health care providers, as well as researchers perceived that the website increased their access to SCI evidence. Access to SCIRE not only improved knowledge of SCI evidence but helped inform changes to the health providers’ clinical practice and improved their confidence in treating SCI clients. The SCIRE information directly influenced the health providers’ clinical decision making, in terms of choice of intervention, equipment needs, or assessment tool.

**Conclusions:**

A Web-based knowledge resource may be a relatively inexpensive method to increase access to evidence-based information, increase knowledge of the evidence, inform changes to the health providers’ practice, and influence their clinical decision making.

## Introduction

eHealth has been defined as “health services and information delivered or enhanced through the Internet and related technologies” [1]. Online health information was one of the earliest (and remains one of the most frequent) applications of eHealth [2,3]. A population-based European study (over 14,000 participants surveyed) estimated that over 50% of adults use the Internet as a source of consumer health information [4]. More recently, health care professionals have increased their use of the Internet to access medical and health information to update their practice and ultimately improve the outcomes of their patients [3]. While free access to academic electronic databases (eg, PubMed) has increased over the years, health care providers have reported numerous barriers to their use, including inability to access the full text of the articles, lack of skills in searching and appraising the literature, and lack of time to compile all of the relevant evidence [5-7]. The amount of information to answer any one clinical question can be massive, and the literature is often conflicting with various levels of quality. For example, a search on the narrow topic of the effectiveness of anticonvulsant medication for the management of pain following spinal cord injury (SCI) results in 13 studies ranging from randomized controlled trials to pre-post studies and retrospective chart reviews [8].

Formal assessments of the body of scientific evidence are an important and time-saving resource for clinicians wishing to incorporate evidence into their clinical decision making and are a key element of putting knowledge into action (ie, knowledge translation) [9]. The Knowledge-to-Action Cycle is a conceptual framework that builds on more than 30 planned action theories and comprises a dynamic process between a knowledge creation and a knowledge application domain [10]. Three levels or “generations” of information have been identified within the knowledge creation domain [11]. Graham et al [11] refer to the information associated with numerous primary studies of varied quality as the “first generation knowledge” or “knowledge inquiry” stage. Synthesized literature may overcome some of the barriers to compiling and weighting relevant literature [12]. This synthesized or aggregated literature often takes the form of a systematic review or meta-analysis and is referred to as “second-generation knowledge” or “knowledge synthesis” [11]. “Third-generation knowledge” or “knowledge tools” take the knowledge synthesis one step further and present user-friendly information with the intent of influencing practice [11]. These include tools and knowledge products such as practice guidelines, care pathways, or websites that synthesize and provide expert commentary on the literature with regard to current practice [11]. Knowledge tools are often preferred by clinicians as they provide explicit, evidence-based descriptions of the benefits and risks of an intervention at the level of the user [13].

Health providers have long cited that having the evidence in one place would facilitate the translation of research to practice [14]. Several websites targeted at frontline health care providers serve as a knowledge product to compile, synthesize, and update selected literature and to provide expert commentary over a focused topic area (eg, specific disease). For example, the Rehabilitation Measures website assists clinicians in their outcome measure selection by reviewing the psychometric properties of outcome measures and providing instructions for their administration in the rehabilitation field [15], while the disease-specific Stroke Engine website critically appraises stroke-related treatments and outcome measures [16]. Profession-specific sites such as the American College of Physicians Journal Club are designed to filter the literature and deliver expert summaries of articles that warrant immediate attention by physicians in internal medicine [17]. Initially disseminated through a traditional journal format, the content is now complemented with an online version.

Access to timely quality evidence is the first step to rectifying the consistent failure to translate research into practice [18] and reducing the unacceptably long time it takes to translate this evidence [19]. While it is known that delivery of specific Internet-based learning activities that require formal registration and participation (eg, virtual anatomy course, teleconference on a specific client case) can have positive effects on educational outcomes for health care providers and students [20], it is not known whether ongoing access to a broad-based Internet knowledge resource can influence the practice of health care providers. We undertook a mixed methods evaluation of the Spinal Cord Injury Rehabilitation Evidence (SCIRE) Project website to answer this question.

The SCIRE Project is an online resource that provides a synthesis of the evidence underlying SCI rehabilitation interventions and is designed to enable relevant decision making in practice settings [21] and public policy. The target audience are health care professionals, researchers, and policy makers. SCIRE uses transparent methods to synthesize all levels of evidence (from randomized controlled trials to case reports), as well as present an overview of relevant outcome measures (eg, purpose of measure, psychometric properties). Over 70 clinicians and researchers with a high degree of expertise in the topics provide summaries of the evidence with implications for practice and decision making.

**Figure 1:**
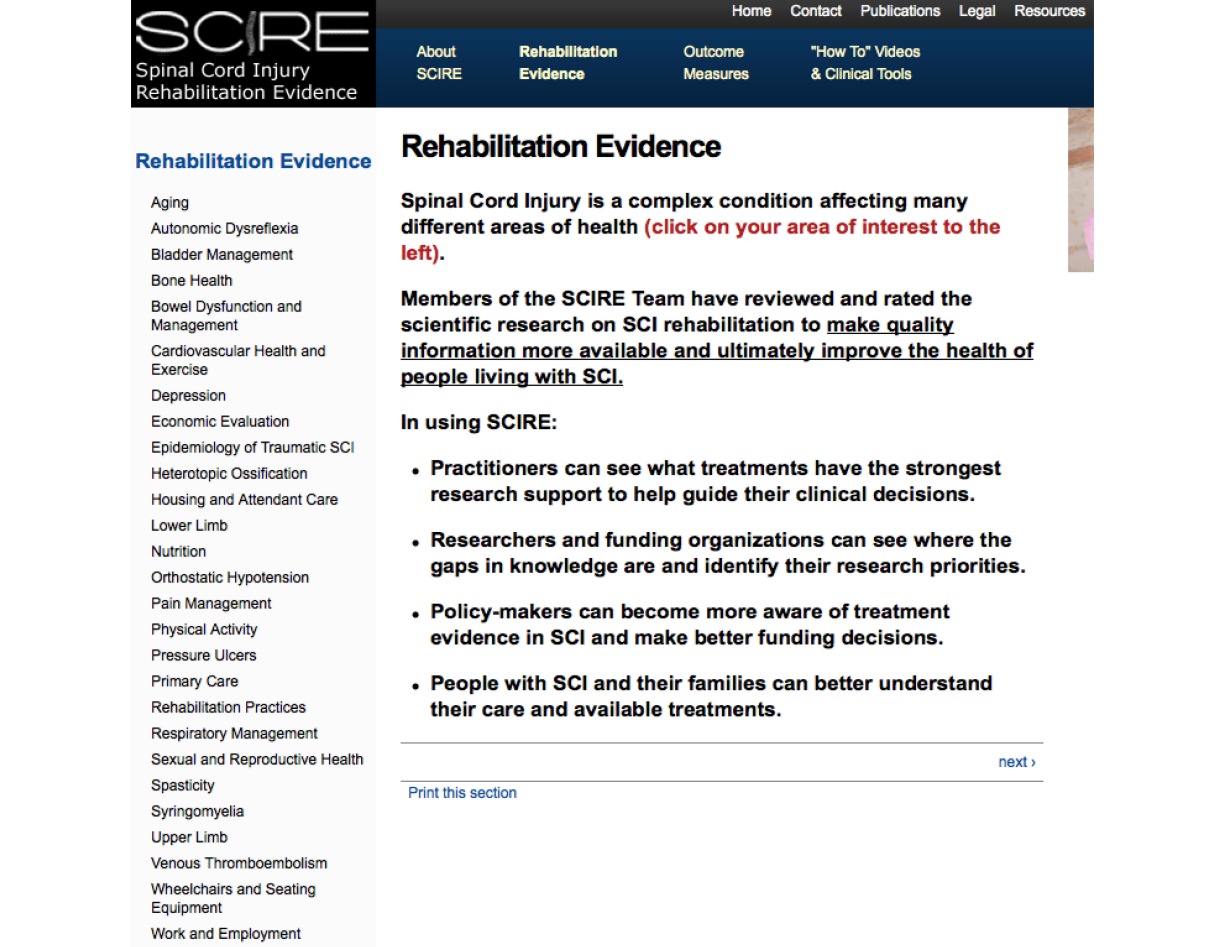
Screenshot illustrating the 27 sections providing rehabilitation evidence related to spinal cord injury. Each section includes background information on the topic, tables of relevant individual studies, a discussion synthesizing the data, a summary of the levels of evidence, and a summary of key points.

**Figure 2:**
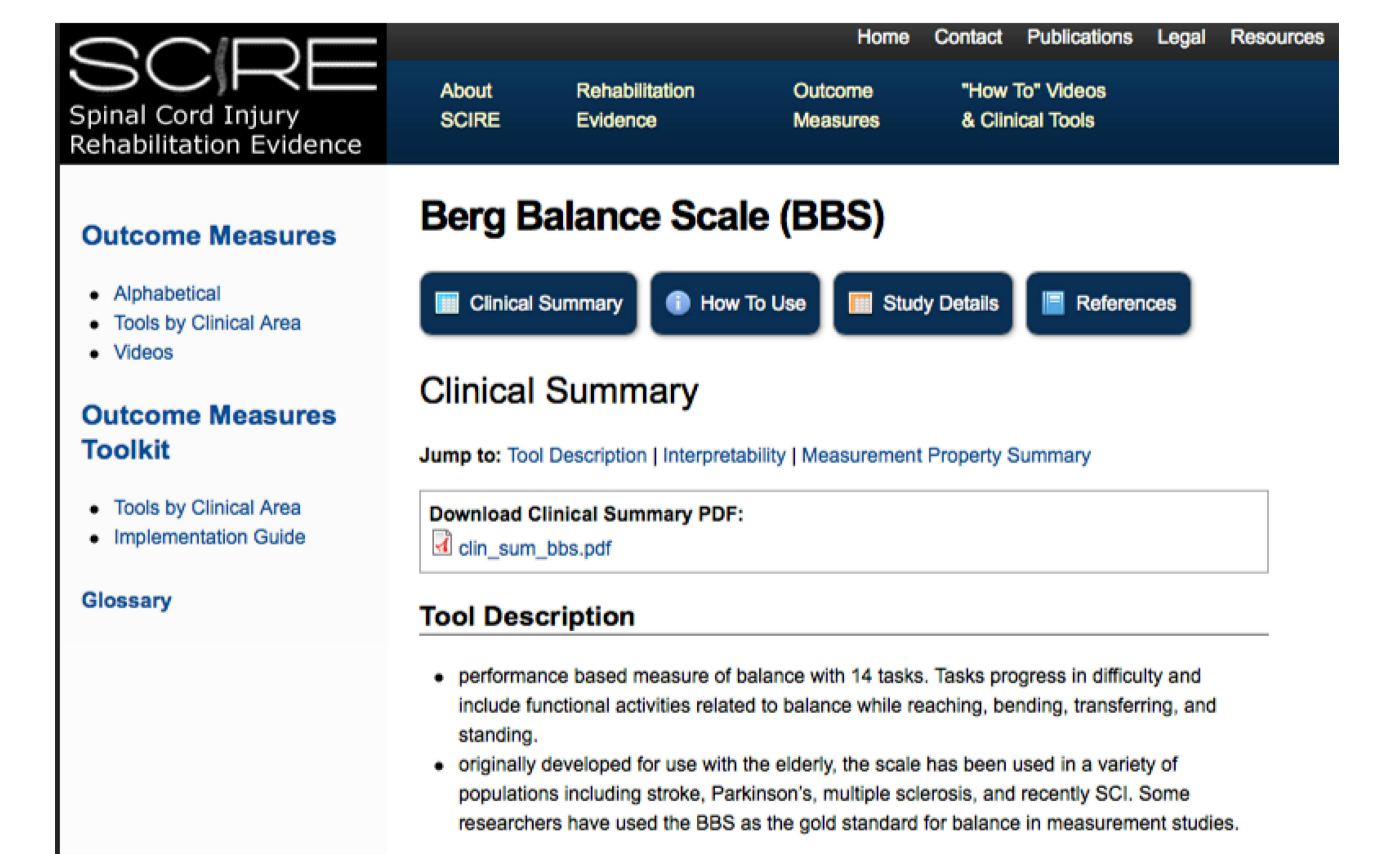
Screenshot illustrating one of the available 105 outcome measures related to spinal cord injury. For each outcome measure, a clinical summary and how-to-use section is provided.

The SCIRE Project consists of two main sections. The first is an overview of the rehabilitation evidence for 27 different sections. The specific sections are illustrated in Figure 1. Each section includes background information on the topic, tables of relevant individual studies, a discussion synthesizing the data, a summary of the levels of evidence, and a summary of key points. Standardized search methods and assignment of the levels of evidence are undertaken to ensure that the methods are replicable and transparent. The second section is an overview of 105 different outcome measures related to spinal cord injury. For each outcome measure, a clinical summary including the psychometrics (reliability, validity) and how-to-use section are provided. One outcome measure is illustrated in Figure 2.

The SCIRE Project is relevant to the knowledge creation domain of the knowledge-to-action cycle and creates “third-generation knowledge” or “knowledge tools” to convey user-friendly information with the intent of influencing practice. Numerous gaps have been identified between current practice and best practice in SCI rehabilitation including the underutilization of standardized outcome measures [22], as well as the lack of implementation of the best evidence for SCI-related complications such as urinary tract infections [23] and pressure ulcers [24]. The SCIRE Project has been online since December 2009.

Our objective was to evaluate the impact of the SCIRE Project on the uptake of knowledge. We hypothesized that this Web-based source of information would (1) increase access to evidence-based information for health care providers and researchers and (2) influence the self-report practice of these health care providers in the management of their clients.

## Methods

### Overview

A 4-part mixed methods evaluation was used to evaluate the SCIRE Project. The protocol was approved by the local university ethics board, and participant consent was required for the surveys and focus groups. An interdisciplinary project advisory team of 12 researchers and clinicians in the field of SCI rehabilitation, including the professions of medicine, health care administration and evaluation, physical therapy, occupational therapy, and research provided input to the development of the tools (surveys and interview guides). To assess the SCIRE Project as a knowledge translation tool, questions were developed using Pathman’s Awareness-to-Adherence Model [25]. Survey instruments were developed using Dillman’s Tailored Design Method [26]. Two re-iterations of each tool were reviewed by the project advisory team beyond the initial version to test logic and functionality. Our study is a first step in determining whether ongoing access to a broad-based Internet knowledge resource can influence the practice of health care providers and utilizes self-report measures that are useful to reveal behaviors and perceptions of clinicians. However, we acknowledge that such measures are subject to various forms of bias (eg, recall bias, social desirability bias).

### Part 1: Website Traffic

We monitored website traffic and utilization using Google Analytics from the initial hosting of the website (December 2009) to July 2012. The purpose was to analyze the website visits and visitor behavior to provide a picture of activity on the website.

### Part 2: Website Survey

A brief (5 minutes) online survey was hosted on the SCIRE Project website over a 5-week period (July 20-Aug 24, 2012) and appeared when visitors exited the website. The purpose was to supplement the Google Analytics data with an 11-question online survey of which 4 questions related to demographics, 1 question on how they heard about SCIRE, 5 questions on use and usability (including navigation; not reported in this paper), and 1 question on impact (the focus of this paper). The question on impact queried the impact of the SCIRE Project on the participant’s access to SCI information and on their clinical practice rated on a 5-point Likert scale.

### Part 3: Targeted End-User Survey

The purpose of the end-user survey was to provide an indication of the awareness of the SCIRE Project, and the impact on clinical or research practice. In contrast to the website survey, participants were health care providers or researchers confirmed to work in the field of SCI rehabilitation. The SCIRE Project was developed with the health provider as the main end-user; however, it is recognized that researchers also constitute a major end-user. A sample of convenience was generated by sending the survey link to 22 coordinators associated with SCI rehabilitation centers in Canada who disseminated it to frontline clinicians who worked in SCI rehabilitation (July 26-Sept 7, 2012). An additional 95 surveys were sent to names gathered from Canadian public health care registries when they specified the interest of the provider to be SCI.

The targeted end-user survey queried the demographics of the individual, reasons why they visited the SCIRE Project, how useful was the SCIRE Project to their information needs, and questions related to the access to information and impact on practice rated on a 5-point Likert scale. Descriptive analyses were used to tabulate the frequency counts to summarize the data for the targeted end-user survey, as well as the website survey.

### Part 4: Focus Groups

Online focus groups with health care providers were undertaken to explore how the SCIRE Project website information is applied in the clinical setting. The interview guide included questions on experience with the SCIRE Project website, motivations for using the SCIRE Project, and extent to which the SCIRE Project has impacted practice. Inclusion criteria for participation in the focus groups were health care providers who had used the SCIRE Project previously. They were selected from those in Part 3 who had agreed to be contacted for a focus group. Two online focus groups (n=8, n=7) were conducted and were led by a trained facilitator with technical support. Each focus group session was recorded and transcribed verbatim. Qualitative content analysis was then applied to the transcripts [27]. The transcripts were read and reviewed separately by 2 researchers. The text was coded into meaning units (statements that relate to the same central meaning), and then the codes were grouped into categories (grouping of codes) until no new categories emerged. Categories were then aggregated into themes (concepts that cut across codes and categories and contributed to our understanding of the impact of the SCIRE Project), and relationships among these were determined. Representative quotes were used to illustrate the themes and to assist the readers in judging the transferability of the findings.

## Results

### Overview

A total of 332 people participated in the evaluation across the website survey, end-user survey, and focus groups.

### Part 1: Website Traffic

A total of 195,519 unique visitors accessed the website between December 2009 and July 2012. The majority of website visitors were from (in descending order) United States, Canada, United Kingdom, Australia, and India. The majority of website visitors (77.62%) were new visitors, while 22.38% were returning visitors. Of the top five most visited content pages, four were specific outcome measures (Functional Independence Measure, Symptom Checklist, Jebsen Hand Test, Hospital Anxiety and Depression Scale), while one was a page within the Respiratory Interventions Chapter (Intermittent Positive Pressure Breathing). Content pages involving the outcome measures were viewed 58.56% of the time, while content from the intervention chapters were viewed the other 41.44% of the time.

### Part 2: Website Survey

There were 171 individuals who completed the website survey. While 26 countries participated, the majority of responses were from the United States (29.8%, 51/171), Canada (29.8%, 51/171), and Australia (14.0%, 24/171). The respondents were primarily health care providers (59.1%, 101/171), while researchers (26.37%, 45/171), SCI consumers and their families (8.2%, 14/171), policy makers (1.7%, 3/171), and others (4.7%, 8/171) made up the remaining groups. Of the health care providers, the majority were physical therapists (41.6%, 42/101), physicians (23.8%, 24/101), occupational therapists (11.9%, 12/101), and nurses (10.9%, 11/101). The health care providers worked in rehabilitation settings (72.3%, 73/101), community settings (13.9%, 14/101), acute settings (7.9%, 8/101), and other settings (5.9%, 6/101) and had a mean of 6.5 (SD 7.6) years of clinical experience. Researchers had a mean of 5.3 (SD 7.2) years of research experience. Most of the health care provider respondents first heard about SCIRE from an Internet search engine result (46.5%, 47/101), referral from a colleague (31.7%, 32/101), published article (10.9%, 11/101), or conference/workshop (10.9%, 11/101).

The responses of the health care providers (n=101) suggested that the website may have had positive impact on practice (Table 1): 89% agreed or strongly agreed that the SCIRE Project had increased their access to SCI evidence, 91% agreed or strongly agreed that the SCIRE Project had improved their knowledge of SCI evidence, 81% agreed or strongly agreed that the SCIRE Project had helped inform changes to their clinical practice, and 69% agreed or strongly agreed that the SCIRE Project had improved their confidence in treating SCI clients.

The researchers agreed or strongly agreed (82-87%) that the SCIRE Project had increased their access to SCI evidence, improved their knowledge of SCI evidence, and supported their current research activities.

**Table 1:** Website survey results showing impact of SCIRE on health care providers and researchers (101 health care providers and 45 researchers completed the survey, but some marked an item as not applicable and were not included in the percent calculation, eg, a clinician who had no research responsibilities for item 2).

		n	Strongly agree, %	Agree, %	Undecided, %	Disagree, %	Strongly disagree, %
**Health care providers**							
	SCIRE has improved my knowledge of SCI evidence	98	56.1	34.7	7.1	1.0	1.0
	SCIRE supports my current research activities	86	41.8	41.8	14.0	1.2	1.2
	SCIRE has helped inform changes to my clinical practice	92	29.3	52.2	15.2	2.2	1.1
	SCIRE has increased my confidence in treating SCI clients	90	29.9	38.9	24.4	5.6	1.1
	SCIRE has increased my access to SCI evidence	97	58.8	29.9	10.3	0	1.0
	SCIRE has not impacted my clinical practice or research activities	98	3.1	5.1	12.2	48.0	31.6
**Researchers**							
	SCIRE has improved my knowledge of SCI evidence	43	44.3	42.0	13.7	0	0
	SCIRE supports my current research activities	39	43.6	38.5	15.4	2.5	0
	SCIRE has increased my access to SCI evidence	39	59.0	28.2	12.8	0	0
	SCIRE has not impacted my research activities	35	0	0	20.0	45.7	34.3

### Part 3: Targeted End-User Survey

Of the 146 targeted end-users, the majority of responses resulted from the distribution to the 22 rehabilitation centers (n=111) and the remaining (n=35) from the direct email request. The majority were working in Canada (83.6%, 122/146), with a small proportion (12.3%, 18/146) from the United States and from other countries (4.1%, 6/146). Due to privacy issues, we were not able to directly email the staff at the rehabilitation centers, and thus do not have information about the response rate for this group. Of the 95 direct email requests, 64 opened the email, and 35 completed the survey.

Of the targeted end-users, 69.9% (102/146) were aware of the SCIRE Project. For the users that were aware of the SCIRE Project, 52.0% (53/102) worked in a rehabilitation setting, 30.4% (31/102) in an acute setting, 15.7% (16/102) in a community setting, and 2.0% (2/102) indicated “other”. The respondents were primarily health care providers (66.7%, 68/102), while researchers (22.5%, 23/102) made up the second largest proportion. Of the health care providers, there was representation from physical therapists (32%, 22/68), physicians (16%, 11/68), occupational therapists (10%, 7/68), nurses (24%, 16/68), psychologists (4%, 3/68), and social worker/counselors (4%, 3/68). Health care providers had a mean 8.5 (SD 7.1) years of experience while researchers had 11.5 (10.8) years of experience. The users that were not aware of the SCIRE Project (30.1%, 44/146) had a similar mix of health providers; however, a larger proportion (52%, 23/44) worked in an acute setting.

Of the users who were aware of the SCIRE Project, 62 health care providers and 31 researchers had accessed the website previously, and their feedback is shown in Table 2. Similar to the website survey, the health care providers indicated positive impact of the website on practice (Table 2): 88% agreed or strongly agree that the SCIRE Project had improved their knowledge of SCI evidence, 93% agreed or strongly agreed that the SCIRE Project had increased their access to SCI evidence, 66% agreed or strongly agreed that the SCIRE Project helped inform changes to practice, and 62% agreed or strongly agreed that the SCIRE Project increased their confidence in treating SCI clients.

Of the researchers, a majority (70-81%) agreed or strongly agreed that the SCIRE Project had increased their access to SCI evidence, improved their knowledge of SCI evidence, and supported their current research activities.

**Table 2:** Targeted user survey results showing impact of SCIRE on health care providers and researchers (62 health care providers and 31 researchers were surveyed, but some marked the item as not applicable and were not included in the percent calculation, eg, a clinician who had no research responsibilities for item 2).

		n	Strongly agree, %	Agree, %	Undecided, %	Disagree, %	Strongly disagree, %
**Health care providers**							
	SCIRE has improved my knowledge of SCI evidence	59	47.1	41.5	9.4	1.9	0
	SCIRE supports my current research activities	51	13.0	58.7	26.1	0	2.2
	SCIRE has helped inform changes to my clinical practice	56	31.4	37.2	25.5	5.9	0
	SCIRE has increased my confidence in treating SCI clients	58	30.8	36.5	26.9	5.8	0
	SCIRE has increased my access to SCI evidence	61	50.9	41.9	7.2	0	0
	SCIRE has not impacted my clinical practice or research activities	57	21.1	15.4	11.5	34.6	17.3
**Researchers**							
	SCIRE has improved my knowledge of SCI evidence	31	40.0	40.0	16.6	0	3.3
	SCIRE supports my current research activities	30	26.7	56.7	10.0	3.3	3.3
	SCIRE has increased my access to SCI evidence	31	43.3	33.4	13.3	6.7	3.3
	SCIRE has not impacted my research activities	29	25.0	14.3	25.0	14.3	21.5

### Part 4: Focus Groups

A total of 15 people over two online focus groups participated. The majority of participants were from Canada (n=10), while the remaining were from outside Canada (United States: n=3; Brazil: n=1; New Zealand: n=1). All were primarily health care providers in the field of SCI rehabilitation; however, several had some responsibilities in research (n=4) and management (n=3). The health professions represented were physical therapy (n=9), medicine (n=3), psychology (n=1), occupational therapy (n=1), and clinical educator (n=1).

From the focus groups, five major themes emerged: evidence in one place, online access, training tool, decision-making tool, and confirmatory evidence.

### Evidence in One Place

A central theme emerged from a number of comments on how the SCIRE Project provided a comprehensive set of topics relevant to SCI in one place. This “one-stop shopping” was particularly advantageous when initiating a search to answer a new question: “It’s a great resource for me when I’m in a situation where I have questions about anything related to SCI, that’s probably the first place I’d start” [P4, group 2] and “If we’re doing anything new or different from the norm, they like to have a good justification. So we would often give the evidence that the SCIRE website provides” [P6, group 2].

Participants acknowledged that having the synthesized evidence could assist with the huge volume of literature: “Having a resource, you know, having this huge explosion of literature...it’s next to impossible...you’d like to keep up with everything, but it’s next to impossible to keep up with it. So, to have a resource that, like I said earlier...this is my first entry into the literature” [P4, group 2].

In addition to the evidence being in one place, the timeliness of this centralized resource was considered important: “I think it’s a great resource…and just hope that they continue to add new evidence in a timely manner” [P5, group 2] and “It’s nice to have a place that you can go that is comprehensive about current information” [P2, group 1].

### Online Access

The online access was viewed as a major advantage of this knowledge tool, especially as it filled a unique niche for a health condition that had a relatively small population. The online SCIRE Project website had the advantage of being quick to access, compared to traditional literature searches. However, while there appeared to be good access to the information within specialized SCI units, some thought there was less awareness of this resource in centers that do not regularly treat SCI but could still benefit from this information:

First of all, the fact that it’s an online tool is excellent.

P5, group 1

Honestly, to my knowledge, it’s the only website like that... that exists, and being in the field of neuro and being in the field of rehab, it seems like there are a lot of resources out there for persons working with primarily the stroke population, but not a whole lot out there for spinal cord injuries.

P1, group 2

The reason I don’t use the publications and journal articles is sort of what the two previous people said, that when I’m on that website that’s usually what I’m there for, so I’m using it fairly quickly.

P1, group 1

I know at a rehab hospital here, we obviously know about it. But I know within the province, people in other hospitals that would treat people with SCI would probably have no idea about SCIRE.

P3, group 1

### Training Tool

Health care providers used this knowledge tool to teach others about SCI care, including staff and students. In some cases, the SCIRE Project provided the evidence to justify their current practice:

For educating new staff…it’s a resource.

P2, group 1

When I have a student and I have a SCI patient, it’s a great resource to direct them to.

P5, group 2

When I have students or I’m doing teaching, I’m on it more frequently...But it’s [using SCIRE] usually when…I’m wanting to justify either my clinical practice or in my instruction to students...you want to have the evidence to back up what you’re doing.

P2, group 2

### Decision-Making Tool

The SCIRE Project directly affected the clinical decision making of the health care providers, such as influencing their choice of intervention, patient equipment needs, or type of assessment tool:

A body of knowledge that a clinician can kind of depend on to help with their practice so it’s definitely impacted on the way we justify things. An example, we were reassessing a C4 patient who we’re looking at exercise options. They’re [the physical therapy team] putting together a proposal for an FES [functional electrical stimulation] bike and we’ll be using SCIRE website to help our case to provide evidence towards that.

P6, group 2

It [SCIRE] has influenced our choice of the tool that we decided to use here for pressure ulcer prevention, so it did have a great influence on that.

P7, group 1

I’ve used it clinically in terms of, for pressure ulcers…find out more about whether electrical stimulation should be used to heal pressure ulcers. So I’ve used that and spread it [the intervention] around to my colleagues.

P6, group 1

I went to the website to look at the literature related to bone density and fracture risk…for clinical purposes before getting people up and trying to get them to walk.

P4, group 2

### Confirmatory Evidence

Several participants suggested that the SCIRE Project content served to confirm what they were already doing in practice and reinforced their understanding of the SCI evidence, rather than impacting their initial decision-making process:

It hasn’t had a huge impact on my practice...I go there less than monthly...it’s actually confirmed what I had read through other journal articles and what colleagues were saying.

P3, group 2

It has been reinforcing in terms of if we’re uncertain, in ensuring that we’re doing what we should be doing.

P6, group 1

So it [SCIRE] didn’t bring any sort of new revolution with relations to practice.

P2, group 1

## Discussion

### Principal Results

Taken together, the quantitative and qualitative findings of this study provide evidence that a Web-based knowledge tool may be effective in disseminating evidence-based practice, informing changes to practice, and bridging the gap between research and practice in SCI rehabilitation. The users were primarily physical therapists, physicians, occupational therapists, and nurses, which suggests that the website attracted health care providers of an interdisciplinary nature. Physical therapists were the largest group who accessed the website possibly due to the large number of interventions and outcome measures included within the SCIRE Project, which related to motor functions. Alternatively, perhaps insufficient promotion has been undertaken with other health provider groups. For example, the SCIRE Project has many interventions and outcome measures dedicated to psychosocial and community aspects of care, which would have major implications for psychologists and social workers, but these providers were underrepresented in the samples.

While the targeted end-user survey indicated that a substantial proportion (70%) were aware of the SCIRE Project resource, some indicated in the focus groups that the SCIRE Project was less well known outside of major rehabilitation or SCI centers. It is a challenge to promote the website to health care providers for whom SCI rehabilitation is a less regular part of their responsibilities (eg, family physician, community social worker). However, these providers may be most likely to benefit from the content for the small number of SCI patients they treat because they do not have the in-house support from other clinicians with SCI experience.

The focus group discussions revealed that the online aspect was an important feature that permitted fast access to the information. The online format allowed the content for a relatively specialized field to have far reach (eg, 26 countries and over 6500 users per month). The website survey and targeted end-user survey confirmed that health care providers, as well as researchers perceived that the website increased their access to SCI evidence.

The focus groups indicated that a major advantage of the SCIRE Project website was that the content was comprehensive and timely and provided one-stop shopping for the best evidence. Users could access the information when it was convenient for them. While participants accessed both the intervention and outcome measure information, the website statistics showed a heavier access to the outcome measures. A number of studies have noted the lack of standardized measures utilized within rehabilitation [28,29]; the website provides one tool to help clinicians select appropriate outcome measures for their practice.

### Impact on Practice

As intended, the SCIRE Project activities are focused on the knowledge creation domain and have a less direct role in the implementation activities of the knowledge-to-action process. However, users across all parts of the evaluation reported not only accessing the evidence but also applying the SCIRE Project evidence to their own practice contexts. Thus, access to the SCIRE Project not only improved knowledge of SCI evidence but helped inform changes to their clinical practice and improved their confidence in treating SCI clients. While all parts of the knowledge-to-action process are important, a recent systematic review showed that in the field of rehabilitation, the most common barrier to the implementation and use of evidence-based medicine was related to research utilization (eg, conflicting results, understanding statistics, literature not compiled in one place, implications for practice not being made clear) [30]. The SCIRE Project provides one important facilitator to enhancing research utilization. The SCIRE Project information directly influenced the health care provider’s clinical decision making, in terms of choice of intervention, equipment needs, or assessment tool.

Some participants felt that the SCIRE Project information served more to confirm or reinforce what they were already doing in practice, rather than impacting their initial decision-making process. Researchers in other areas have noted that guidelines provide accountability in the eyes of other stakeholders [31] and may improve the clinician’s confidence as it legitimizes their approach [32]. In some instances, it appeared that some health care providers accessed the SCIRE Project to justify their practice to others, especially students. The finding that users were accessing the SCIRE Project to teach others suggests a snowball effect in terms of its dissemination and impact. It is likely that to change practice, the optimal use of the SCIRE Project would be to use it in conjunction with active (eg, audit and feedback) and multicomponent implementation strategies [12,33] to facilitate uptake and change in practice. Moreover, it may be that resources like the SCIRE Project represent a platform on which to develop even more focused tools, such as clinical decision support systems that reflect customized and contextualized approaches to facilitate clinical decision making.

### Limitations

As we examined the natural uptake of this website information, no control group was used to control for biases such as social desirability within the self-report responses. Furthermore, due to the fact that the samples were of convenience, they may not have been representative samples, and it is possible that those who perceived to have gained benefits from the SCIRE Project would be more likely to participate in the surveys and focus groups. The website was relatively inexpensive to develop and maintain and did not include more interactive attributes such as Web 2.0 blogs or wikis, which require more monitoring but could potentially develop a strong collaborative community of users.

### Conclusions

Most notably, these findings demonstrate that the SCIRE Project was effective in disseminating information about evidence-based practice to an interdisciplinary audience and that there was some indication that it facilitated practice changes in the primary target audience of frontline clinicians. Information relating to outcome assessment represented the most frequently sought materials. It was noted that the online nature of the SCIRE Project, coupled with its comprehensiveness and timeliness, facilitated utilization as a one-stop resource for informing practice. However, there were suggestions that this resource also served to confirm or provide a rationale for existing practice, which reflects the nature of the SCIRE Project as a compilation of evidence. Additionally, there was some evidence that the SCIRE Project may have had less impact outside of major rehabilitation and SCI centers.

Taken together, these findings suggest that it might be beneficial to more explicitly describe and demonstrate to users how they might take full advantage of the SCIRE Project—not only as a knowledge creation resource (ie, evidence synthesis) but also by providing examples of how users might employ this or similar resources in their knowledge-to-action (ie, implementation) activities.

## Abbreviations

SCIRE: Spinal Cord Injury Rehabilitation Evidence
SCI: Spinal cord injury
